# Radioiodinated Bicyclic RGD Peptide Derivatives for Enhanced Tumor Accumulation

**DOI:** 10.3390/ph18040549

**Published:** 2025-04-08

**Authors:** Naoya Kondo, Marika Kato, Aoi Oshima, Fuko Hirano, Anna Miyazaki, Takashi Temma

**Affiliations:** 1 Department of Biofunctional Analysis, Graduate School of Pharmaceutical Sciences, Osaka Medical and Pharmaceutical University, 4-20-1 Nasahara, Takatsuki 569-1094, Osaka, Japan; kondon@hirakata.kmu.ac.jp (N.K.); anna.miyazaki@ompu.ac.jp (A.M.); 2 Division of Fundamental Technology Development, Near InfraRed Photo-ImmunoTherapy Institute, Kansai Medical University, 2-5-1 Shin-machi, Hirakata 573-1010, Osaka, Japan

**Keywords:** integrin, bicyclic peptide, albumin, dimerization

## Abstract

**Background/Objectives**: Integrin α_V_β_3_ plays a crucial role in tumor angiogenesis and cancer progression, making it a key target for radiolabeled probes used in imaging and therapy. A previously developed probe, [^125^I]bcRGD, exhibited high selectivity for α_V_β_3_ but limited tumor accumulation due to rapid blood clearance. This study aimed to address this issue through two strategies: (1) conjugating albumin-binding molecules to enhance systemic circulation and (2) dimerizing RGD peptides to improve binding affinity via multivalency effects. **Methods**: Three [^125^I]bcRGD derivatives were synthesized: [^125^I]bcRGD_pal_ (with palmitic acid), [^125^I]bcRGD_iba_ (with 4-(*p*-iodophenyl)butyric acid), and [^125^I]bcRGD_dimer_ (a dimeric bicyclic RGD peptide). Their physicochemical properties, α_V_β_3_-selectivity, albumin-binding capacity, and biodistribution were assessed in vitro and in vivo using tumor-bearing mice. Tumor models included α_V_β_3_-high U-87 MG and α_V_β_3_-low A549 xenografts. **Results**: [^125^I]bcRGD_pal_ and [^125^I]bcRGD_iba_ exhibited prolonged blood retention (30-fold and 55-fold vs. [^125^I]bcRGD, respectively) and increased tumor accumulation (3.9% ID/g and 3.6% ID/g at 2 h, respectively). Despite improved systemic circulation, tumor-to-blood ratios remained low (<1), indicating limited tumor retention. [^125^I]bcRGD_dimer_ achieved significantly greater tumor accumulation (4.2% ID/g at 2 h) and favorable tumor-to-blood (22) and tumor-to-muscle (14) ratios, with a 5.4-fold higher uptake in U-87 MG tumors compared to A549 tumors. **Conclusions**: Dimerization was more effective than albumin binding in enhancing bcRGD’s tumor-targeting potential. The dimeric probe demonstrated improved tumor accumulation, favorable pharmacokinetics, and preserved integrin selectivity. These findings provide a foundation for further structural optimization of bicyclic RGD peptides for integrin α_V_β_3_-targeted imaging and therapy applications.

## 1. Introduction

Integrin α_V_β_3_ plays a pivotal role in tumor angiogenesis, as well as in cancer cell adhesion, migration, and invasion [1,2]. It is highly expressed on tumor endothelial cells, contributing to cancer progression and metastasis [3]. Overexpression of α_V_β_3_ has been observed in various cancers, including breast, pancreatic, and cervical cancers, with expression levels correlating with tumor grade and prognosis [4,5].

Cyclic RGD peptides (cRGDs) are recognized ligands for integrin α_V_β_3_ [6], leading to the development of numerous radiolabeled imaging probes [7,8,9]. Radiolabeled cRGD peptides are anticipated to play an important role in nuclear medicine imaging for the detection of α_V_β_3_ and the evaluation of angiogenesis in cancer. Assessing cancer angiogenesis can aid in predicting the efficacy of angiogenesis inhibitors and in providing a more detailed understanding of cancer pathology [10,11]. However, cRGDs exhibit cross-reactivity with alternative integrin subtypes [10], particularly α_V_β_5_, where structural homology impedes selective α_V_β_3_ ligand development [12]. For instance, the inhibitor cilengitide, which is derived from the cRGD peptide and has undergone clinical investigation, inhibits both α_V_β_3_ and α_V_β_5_ [13]. Since integrins α_V_β_3_ and α_V_β_5_ are expressed in distinct regions within cancer tissues and play different biological roles, probes capable of distinguishing between these subtypes could greatly improve tumor characterization, prognosis prediction, and the development of targeted therapies [14,15,16]. This unmet need has driven the development of α_V_β_3_-specific peptides [12,17] and engineered knottin-RGD constructs with enhanced binding kinetics [18].

Recent advances yielded a bicyclic RGD peptide (bcRGD) exhibiting remarkable α_V_β_3_ selectivity [19]. The binding potency of bcRGD to α_V_β_3_ was comparable to that of cyclo-(RGDfK), a classical cyclic RGD; however, the α_V_β_3_/α_V_β_5_ selectivity ratio (ratio of corresponding IC_50_ values) was ≤0.003, significantly outperforming cyclo-(RGDfK) (0.2), cilengitide (4.7), and knottin-RGD (0.5) [19]. A radiolabeled probe based on this bicyclic peptide, [^125^I]bcRGD, was synthesized and shown to selectively accumulate in α_V_β_3_-expressing cancer cells and xenograft models in mice [20]. However, like many low-molecular-weight peptides, [^125^I]bcRGD exhibited rapid blood clearance [21], resulting in minimal tumor accumulation in α_V_β_3_-expressing tumors 120 min post-administration [20]. It was hypothesized that structural modifications to [^125^I]bcRGD, aimed at enhancing tumor accumulation, could significantly advance integrin α_V_β_3_-targeted imaging and support the development of nuclear medicine therapeutics.

**Figure 1:**
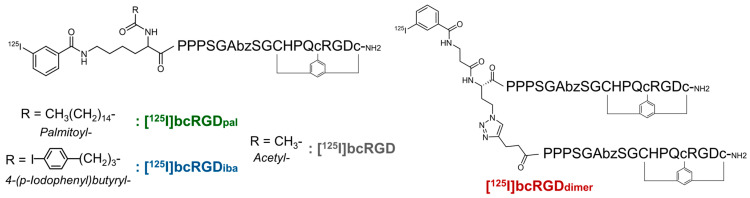
Schematic representation of radioiodinated bcRGD derivatives. Structure and color correspond in all figures; [^125^I]bcRGD_pal_ (green), [^125^I]bcRGD_iba_ (blue), [^125^I]bcRGD_dimer_ (red), and [^125^I]bcRGD (gray).

This study aimed to enhance tumor accumulation by employing two strategies. The first focused on prolonging blood retention, as extended systemic circulation increases radiopharmaceutical distribution to tumors. Incorporating albumin-binding molecules is a well-established approach for improving tumor accumulation by extending peptide half-life in circulation [22,23]. We selected 4-(*p*-iodophenyl)butyric acid (IBA) and palmitic acid (PAL), extensively studied as albumin-binding small molecules [24,25,26,27], and designed two [^125^I]-labeled peptides, [^125^I]bcRGD_iba_ and [^125^I]bcRGD_pal_, with IBA or PAL at the N-terminal, whereas bcRGD retained an acetyl group (Figure 1 and Figure S1).

The second strategy utilized peptide dimerization to exploit the multivalency effect, where multiple binding sites significantly enhance receptor-binding affinity [28]. This approach has been shown to improve cancer cell targeting, with multimeric RGD peptides exhibiting superior binding affinity compared to monomeric counterparts [29,30]. In this study, bcRGD was modified at its N-terminal to introduce either an alkyne or azide group. Using a click reaction, a radiolabeled dimeric probe, [^125^I]bcRGD_dimer_, was synthesized, incorporating two bcRGD units (Figure 1 and Figure S1).

This study provides a foundational assessment of these novel radiolabeled probes to evaluate their potential for therapeutic and diagnostic applications targeting integrin α_V_β_3_.

## 2. Results

### 2.1. Properties of Prepared Peptides

All peptides were synthesized with purities exceeding 99%. Electrospray ionization mass spectrometry (ESI-MS) data are presented in Table S1 and Figure S4a–h. Given the absence of other reactive functional groups—except for the ε-amino groups of the N-terminal Lys in bcRGD_pal_ and bcRGD_iba_, and the amino group of the terminal β-alanine in bcRGD_azide_—these peptides were expected to undergo site-specific radioiodination by [^125^I]*N*-succinimidyl 3-iodobenzoate ([^125^I]SIB) (Figure 1). The radiochemical yield (RCY) for [^125^I]bcRGD_pal_ and [^125^I] bcRGD_iba_ from [^125^I]SIB was 59% and 70%, respectively, with radiochemical purities (RCP) exceeding 99% following high-performance liquid chromatography (HPLC) purification. The RCY for [^125^I]bcRGD_azide_ from [^125^I]SIB was 40% with RCP > 99%, while the RCY for [^125^I]bcRGD_dimer_ from [^125^I]bcRGD_azide_ was 61% (total 24% from [^125^I]SIB) with RCP > 99%. [^125^I]bcRGD_pal_ (retention time (T_R_) = 18.2 min, condition A), [^125^I]bcRGD_iba_ (T_R_ = 20.4 min, condition B), and [^125^I]bcRGD_dimer_ (T_R_ = 12.2 min, condition C). Each was fully separated from its unreacted precursor (bcRGD_pal_, T_R_ = 13.1 min; bcRGD_iba_, T_R_ = 12.4 min; bcRGD_alkyne_, T_R_ = 10.8 min) by HPLC, allowing all radiolabeled peptides to be obtained in a non-carrier-added form, with molar activities estimated at 81 GBq/µmol.

### 2.2. In Vitro α_V_β_3_ Selectivity

**Figure 2:**
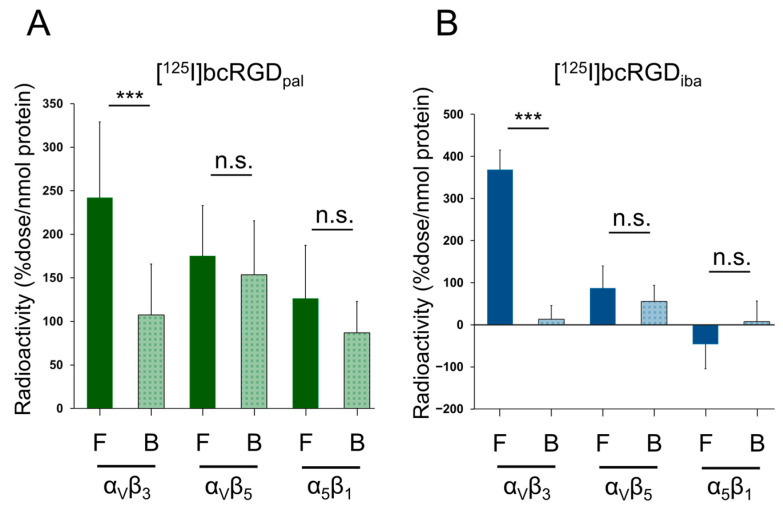
Radioactivity accumulation in α_V_β_3_, α_V_β_5_, and α_5_β_1_ proteins after 2 h of incubation with [^125^I]bcRGD_pal_ (**A**) and [^125^I]bcRGD_iba_ (**B**). F (Free): without cyclo-(RGDfK); B (Blocking): with cyclo-(RGDfK) (25 μM). Data are expressed as mean ± standard deviation and analyzed using an unpaired *t*-test. *** *p* < 0.001 compared to the corresponding blocking group. n.s.: not significant.

Both [^125^I]bcRGD_pal_ and [^125^I]bcRGD_iba_ exhibited high radioactivity accumulation in α_V_β_3_, which was significantly inhibited by the broad-spectrum integrin inhibitory peptide **cyclo-**(RGDfK). No differences were observed in radioactivity accumulation for α_V_β_5_ and α_5_β_1_ between treatment and inhibitor groups, confirming that [^125^I]bcRGD_pal_ and [^125^I]bcRGD_iba_ specifically recognized α_V_β_3_ in vitro (Figure 2).

### 2.3. Octanol–Water Distribution Coefficient (Log D)

**Figure 3:**
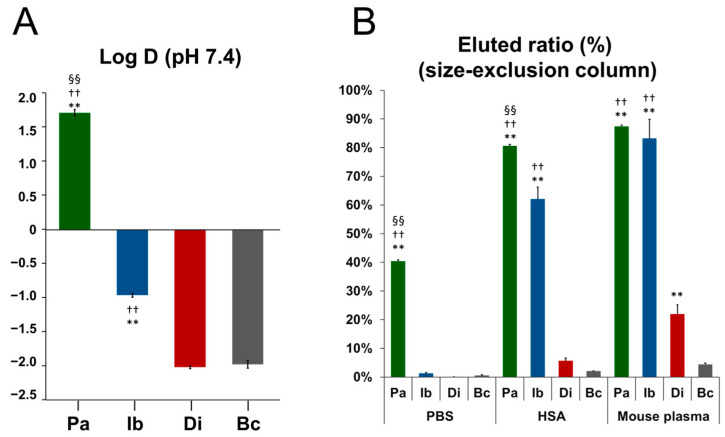
Physicochemical properties of radioiodinated bcRGD derivatives. (**A**) Octanol–water distribution coefficient (log D) determined by radioactivity distribution. (**B**) Radioactivity elution ratio from a size-exclusion column after incubation with PBS, HSA, and mouse plasma. Data are expressed as mean ± standard deviation and analyzed using Tukey’s multiple comparison test. ** *p* < 0.01, compared to Bc, ^††^ *p* < 0.01 compared to Di, ^§§^ *p* < 0.01 compared to Ib. Pa: [^125^I]bcRGD_pal_; Ib: [^125^I]bcRGD_iba_; Di: [^125^I]bcRGD_dimer_; Bc: [^125^I]bcRGD.

The log D values revealed that [^125^I]bcRGD_pal_ (Log D = 1.71) and [^125^I]bcRGD_iba_ (Log D = −0.97) were significantly higher than [^125^I]bcRGD (Log D = −2.02). [^125^I]bcRGD_pal_ exhibited particularly high Log D values, significantly exceeding those of [^125^I]bcRGD_iba_, while [^125^I]bcRGD_dimer_ (Log D = −1.98) showed no significant difference from [^125^I]bcRGD (Figure 3A).

### 2.4. Albumin Binding Property

Elution rates from the size-exclusion resin followed the order: PBS < human serum albumin (HSA) < mouse plasma (Figure 3B). In mouse plasma, [^125^I]bcRGD exhibited a negligible elution rate (2%), whereas [^125^I]bcRGD_pal_ and [^125^I]bcRGD_iba_ displayed high elution rates (>80%). [^125^I]bcRGD_dimer_ showed moderate elution (22%), significantly higher than [^125^I]bcRGD. In HSA, [^125^I]bcRGD_pal_ (80%) had a higher elution rate than [^125^I]bcRGD_iba_ (60%), while [^125^I]bcRGD_dimer_ exhibited a low elution **rate** (6%). In PBS, only [^125^I]bcRGD_pal_ (40%) was eluted, with other peptides showing negligible elution.

### 2.5. In Vivo Study

**Figure 4:**
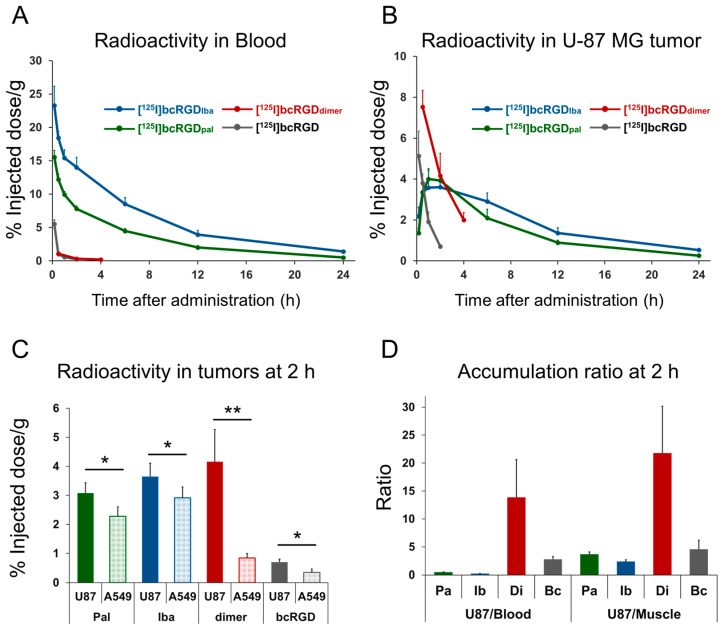
Biodistribution of radioiodinated bcRGD derivatives in tumor-bearing mice following intravenous administration. Radioactivity levels in (**A**) blood and (**B**) U-87 MG tumors. (**C**) Radioactivity distribution in U-87 MG and A549 tumors at 2 h post-administration. Data are expressed as mean ± standard deviation and analyzed using an unpaired *t*-test. * *p* < 0.05, ** *p* < 0.01, compared to the corresponding accumulation in A549. (**D**) U-87 MG-to-blood and U-87 MG-to-muscle radioactivity ratios at 2 h post-administration. Data are presented as mean ± standard deviation.

Immunohistochemical analysis confirmed high α_V_β_3_ expression in U-87 MG cells and higher α_V_β_5_ expression in A549 cells (Figure S2). Biodistribution data for [^125^I]bcRGD_pal_ and [^125^I]bcRGD_iba_ in α_V_β_3_-positive U-87 MG-bearing mice are summarized in Table 1 and Table 2, respectively. Biodistribution data for [^125^I]bcRGD_dimer_ in U-87 MG and A549 co-transplanted mice are presented in Table 3. Temporal comparisons of blood and tumor radioactivity post-administration are illustrated in Figure 4A,B.

Both [^125^I]bcRGD_pal_ and [^125^I]bcRGD_iba_ exhibited increased blood retention compared to [^125^I]bcRGD, with [^125^I]bcRGD_iba_ demonstrating the longest retention. [^125^I]bcRGD_dimer_ did not significantly differ from [^125^I]bcRGD in blood retention. At 2 h post-administration, blood radioactivity levels were 7.8% ID/g for [^125^I]bcRGD_pal_, 14% ID/g for [^125^I]bcRGD_iba_, 0.3% ID/g for [^125^I]bcRGD_dimer_, and 0.3% ID/g for [^125^I]bcRGD. [^125^I]bcRGD_iba_ remained 55 times more radioactive in the blood than [^125^I]bcRGD, highlighting its prolonged systemic circulation.

Regarding tumor accumulation, [^125^I]bcRGD_pal_ and [^125^I]bcRGD_iba_ exhibited significantly higher radioactivity than [^125^I]bcRGD. At 2 h post-administration, tumor-accumulated radioactivity measured 3.9, 3.6, and 0.7% ID/g for [^125^I]bcRGD_pal_, [^125^I]bcRGD_iba_, and [^125^I]bcRGD, respectively. However, the tumor-to-blood radioactivity ratio remained below 1 at all time points for [^125^I]bcRGD_pal_ and [^125^I]bcRGD_iba_. In contrast, [^125^I]bcRGD_dimer_ demonstrated both greater tumor accumulation (4.2% ID/g at 2 h) and superior tumor-to-blood (22) and tumor-to-muscle (14) ratios compared to [^125^I]bcRGD (2.8 and 4.6, respectively). At 2 h post-administration, all probes showed significantly higher accumulation in α_V_β_3_-high expressing U-87 MG tumors compared to low-expressing A549 tumors (Figure 4C,D). Notably, [^125^I]bcRGD_dimer_ exhibited the highest accumulation ratio (U87/A549 = 5.4). With the exception of excretion-related organs, all peptides showed high accumulation in bone and lung, which are known to express integrin α_V_β_3_ [31,32]. Minimal thyroid accumulation across all probes indicated negligible in vivo deiodination.

## 3. Discussion

Integrin α_V_β_3_-targeted probes follow a defined trajectory from the plasma to the extracellular space, where they bind to integrin α_V_β_3_ on tumor cell membranes and are occasionally internalized into tumor cells [33,34]. Enhancing tumor accumulation requires deliberate intervention in this process.

Our initial strategy to increase tumor accumulation focused on raising the concentration of radioactive probes in the plasma. Albumin binders, PAL and IBA, have been extensively explored as agents that prolong peptide circulation in the bloodstream [24,25,26,27]. PAL, a long-chain fatty acid, binds albumin at multiple sites, including subdomains IIIA and IIIB [35], while IBA is structurally optimized to bind tightly to Sudlow site II of HSA [36,37]. In our design, where PAL and IBA were conjugated to the N-terminal of bcRGD, both probes retained specific binding to α_V_β_3_ in vitro and showed significantly higher accumulation in α_V_β_3_-high expressing U-87 MG tumors compared to low-expressing A549 tumors in vivo, confirming preserved α_V_β_3_ recognition (Figure 4C).

A key difference between [^125^I]bcRGD_pal_ and [^125^I]bcRGD_iba_ was their varied hydrophobicity, as reflected by their Log D values. [^125^I]bcRGD_pal_ displayed the greatest lipophilicity (Log D > 1, Figure 3A), which may explain its nonspecific adsorption during in vitro protein-binding assays. This nonspecific interaction reduced the distinction in accumulation between the Free and Blocking groups, as well as between α_V_β_3_ and other integrins (α_V_β_5_ and α_5_β_1_). Additionally, size-exclusion chromatography indicated that [^125^I]bcRGD_pal_ eluted as a high-molecular-weight fraction even in PBS (Figure 3B), suggesting possible self-assembly into larger structures in aqueous environments [38].

As anticipated, [^125^I]bcRGD_pal_ and [^125^I]bcRGD_iba_ showed a substantial increase in blood radioactivity (30-fold and 55-fold, respectively, compared to [^125^I]bcRGD at 2 h post-administration, Figure 4A). Although [^125^I]bcRGD_pal_ exhibited higher protein-binding efficiency in vitro, in vivo data revealed that [^125^I]bcRGD_iba_ achieved greater blood radioactivity retention. While detailed in vivo probe stability was not evaluated in this study, our results confirmed successful albumin binding and improved systemic exposure as designed. Tumor accumulation of both [^125^I]bcRGD_pal_ and [^125^I]bcRGD_iba_ also increased (5.6-fold and 5.1-fold vs. [^125^I]bcRGD at 2 h post-administration, Figure 4B); however, the tumor-to-blood radioactivity ratio remained persistently low. Unlike earlier studies utilizing albumin-binding molecules to improve tumor accumulation, which reported sustained tumor radioactivity even at later time points post-injection [24,25], our findings showed a marked decline in tumor accumulation for [^125^I]bcRGD_pal_ and [^125^I]bcRGD_iba_ at 24 h post-administration (0.3% ID/g and 0.5% ID/g, respectively), suggesting poor tumor retention.

The consistently low tumor-to-blood radioactivity ratio and reduced tumor accumulation at later time points imply that the binding equilibrium between α_V_β_3_ and the probes may favor dissociation, pointing to an insufficient binding affinity for α_V_β_3_. Additionally, limited intracellular radionuclide retention may contribute to this effect. Previous studies employed metallic radionuclides such as ^68^Ga and ^177^Lu [25,27,39], which demonstrated prolonged intracellular retention after internalization. In contrast, our study used non-metallic ^125^I, which is more prone to extracellular efflux than metallic radionuclides [40]. Future research may enhance tumor retention by labeling bcRGD_pal_ or bcRGD_iba_ with metallic radionuclides.

Our second strategy focused on enhancing the probe’s binding affinity to integrin α_V_β_3_. Increased α_V_β_3_ binding affinity is expected to improve tumor accumulation, tumor-to-blood ratio, and α_V_β_3_-specific targeting [41]. Dimerization of integrin-targeted probes is a widely used approach to enhance affinity [42,43,44], and in this study, we explored the dimerization of bcRGD. This strategy ([^125^I]bcRGD_dimer_) resulted in minimal changes in pharmacokinetics but significantly elevated accumulation in α_V_β_3_-positive U-87 MG tumors (Table 3). Importantly, [^125^I]bcRGD_dimer_ demonstrated the highest accumulation ratio in U-87 MG tumors relative to A549 tumors, which exhibit low α_V_β_3_ expression (Figure 4D), indicating that in vivo tumor accumulation correlates with integrin expression levels. In addition to the expected excretory organs (liver, kidneys, and intestine), notable radioactivity accumulation was observed in bone and lung tissues. This is consistent with the high expression of integrin α_V_β_3_ in osteoclasts, where it regulates adhesion, migration, and bone resorption [31,45], as well as in pulmonary microvascular endothelial cells, where it contributes to inflammation and vascular permeability [32]. Under pathological conditions such as pulmonary fibrosis and acute lung injury, integrin α_V_β_3_ is implicated in fibroblast activation and cytoskeletal remodeling—processes that promote tissue stiffening and fibrosis [46]. For [^125^I]bcRGD_dimer_, the lung-to-blood radioactivity ratio was 3.7 at 2 h post-administration, suggesting potential diagnostic value in these disease contexts.

This study represents the first attempt to dimerize bcRGD, resulting in enhanced tumor accumulation. Initially, we proposed a synthetic pathway where the peptide was dimerized via a click chemistry reaction before being radiolabeled with ^125^I; however, low overall yields necessitated a three-step synthesis approach (Figure S3). Optimization of reaction conditions will be crucial for improving yield in future studies. The observed improvement in tumor retention is likely due to an increased binding affinity to integrins. However, attempts to quantify this affinity using Ni-NTA beads with immobilized integrin proteins were unsuccessful, owing to nonspecific adsorption of [^125^I]bcRGD_dimer_ to the beads. Future research should clarify the relationship between bcRGD multimerization and its binding affinity. Competitive inhibition assays between radiolabeled ligands (e.g., radiolabeled cRGD) and unlabeled bcRGD_dimer_ would mitigate nonspecific adsorption interference and facilitate more precise affinity determination. Furthermore, multimerized bcRGD offers multiple avenues for structural refinement. For instance, strategies such as trimerization [29] and tetramerization [47], previously applied to cRGD probes, could be extended to bcRGD. Furthermore, as the spatial arrangement between binding motifs is critical for bivalency, optimizing the linker length remains an important consideration for the bcRGD dimer [48]. Incorporating metallic radionuclides via chelation may offer further benefits, including streamlined radiolabeling [49] and prolonged intracellular retention following internalization [40]. Combining these designs with albumin-binding molecules could further enhance tumor accumulation [50]. It is anticipated that the multimeric bcRGD, which demonstrated promising potential in this study, will evolve into more effective therapeutic and diagnostic probes through future structural refinements.

In conclusion, this study provides a comprehensive assessment of the physicochemical characteristics, pharmacokinetics, and tumor-targeting performance of PAL- and IBA-conjugated peptides, offering valuable insights into the structural engineering of bicyclic peptides for advanced applications. Moreover, this work constitutes the first report on bcRGD dimerization, detailing its pharmacokinetic profile and tumor accumulation behavior. These findings establish a foundation for further structural optimization of bcRGD, paving the way for its use in integrin α_V_β_3_-targeted imaging and therapy. As such, the outcomes of this study may significantly contribute to progress in the diagnosis and treatment of integrin α_V_β_3_-associated diseases.

## 4. Materials and Methods

### 4.1. Preparation of Peptides

All amino acids and coupling reagents were purchased from Watanabe Chemical Industries (Hiroshima, Japan) and used without further purification. The linear peptide sequences—palmitic acid-KPPPSG-Abz-SGCHPQcRGDc-NH_2_ (RGD_pal_; Abz, 4-amino-benzoic acid; c, D-Cys), 4-(*p*-iodophenyl)butyric acid-KPPPSG-Abz-SGCHPQcRGDc-NH_2_ (RGD_iba_), β-ala-γ-azidohomoalanine-KPPPSG-Abz-SGCHPQcRGDc-NH_2_ (RGD_azide_), and 4-pentynoic acid-KPPPSG-Abz-SGCHPQcRGDc-NH_2_ (RGD_alkyne_) were synthesized on Rink amide-PEG resin (A00213, Watanabe Chemical) using Fmoc solid-phase peptide synthesis. Peptides were cleaved from the resin using a cocktail of trifluoroacetic acid (TFA)/phenol/water/triisopropylsilane (88:5:5:2) and purified by reversed-phase HPLC (RP-HPLC) on a C18-reversed-phase column (COSMOSIL 5C18-AR-II, 10 mm ID × 250 mm; Nacalai Tesque, Kyoto, Japan) with UV detection to obtain the amount needed for the experiment. The mobile phase consisted of a linear gradient of 0.1% TFA in water and 0.1% TFA in acetonitrile, applied under three conditions: 70:30 to 10:90 (condition A), 90:10 to 30:70 (condition B), or 90:10 to 50:50 (condition C), over 30 min at a flow rate of 5.0 mL/min.

Purified linear peptides were dissolved in a 1:3 mixture of acetonitrile and water. To this solution, 1.1 equivalents of 1,3,5-tris(bromomethyl) benzene in acetonitrile were added, followed by homogenization. Ammonium carbonate (44 eq. in water) was then introduced, and the mixture was shaken for 60 min at room temperature. Bicyclic RGD peptides (bcRGD_pal_, bcRGD_iba_, bcRGD_azide_, and bcRGD_alkyne_) were subsequently purified via RP-HPLC. To synthesize I-bcRGD_pal_, I-bcRGD_iba_, and I-bcRGD_azide_, N-succinimidyl iodobenzoate (SIB) was prepared according to previous reports [51]. SIB (1.5 eq.) and bcRGD were dissolved in a 1:1 mixture of acetonitrile and borate buffer (0.1 M, pH 8.5), reacted at 40 °C for 1 h, and purified by RP-HPLC. For the synthesis of I-bcRGD_dimer_, equimolar amounts of I-bcRGD_azide_ and bcRGD_alkyne_ were dissolved in dry DMF with CuSO_4_·5H_2_O (50 eq.) and ascorbic acid (100 eq.), stirred for 1 h at room temperature, and purified by RP-HPLC.

The purified peptides were characterized by analytical HPLC under the same conditions as those used for semi-preparative HPLC, as well as by ESI-MS (LCMS-8045; Shimadzu, Kyoto, Japan). bcRGD was also synthesized following our previously reported method [20].

### 4.2. Radiolabeling

Na[^125^I]I was obtained from PerkinElmer Japan (Yokohama, Japan). [^125^I]SIB was prepared using previously described procedures in a no-carrier-added form (estimated molar activity: 81 GBq/μmol) [51]. For radiolabeling, bcRGD_pal_ or bcRGD_iba_ (200 μg) was dissolved in a mixture of borate buffer (100 μL, 0.1 M, pH 8.5) and acetonitrile (100 μL), and then added to [^125^I]SIB (10–20 MBq) dissolved in acetonitrile (100 μL). After 1 h incubation at 40 °C, the reaction mixture was purified by RP-HPLC to yield [^125^I]bcRGD_pal_ or [^125^I]bcRGD_iba_.

To synthesize [^125^I]bcRGD_dimer_, [^125^I]bcRGD_azide_ was prepared using the same procedure as for [^125^I]bcRGD_pal_ and [^125^I]bcRGD_iba_. A reaction mixture consisting of [^125^I]bcRGD_azide_, CuSO_4_·5H_2_O (2.2 mg), ascorbic acid (3.1 mg), and bcRGD_alkyne_ (200 μg) in dry DMF was stirred for 1 h at room temperature, followed by purification via RP-HPLC. The purified radiolabeled peptides were further analyzed by RP-HPLC to assess radiochemical purity.

The final purified solutions of [^125^I]bcRGD_pal_, [^125^I]bcRGD_iba_, and [^125^I]bcRGD_dimer_ were dried under reduced pressure and reconstituted in an appropriate buffer for subsequent experiments. [^125^I]bcRGD was also synthesized according to our previously published method [20]. Detailed synthesis schemes and protocols for [^125^I]SIB and [^125^I]bcRGD_dimer_ are provided in the Supplementary Information (Figure S3).

### 4.3. In Vitro Selectivity Assay

Binding assays were conducted following our previously reported method [20], using the Dynabeads™ His-Tag Isolation and Pulldown protocol (Thermo Fisher Scientific, Tokyo, Japan). Briefly, [^125^I]bcRGD_pal_ or [^125^I]bcRGD_iba_ (10 kBq/sample) was incubated at 37 °C for 2 h in 200 μL of incubation buffer (3.25 mM sodium phosphate, 70 mM NaCl, 0.01% Tween 20, pH 7.4) with 10 pmol of His-tagged integrin α_V_β_3_, α_V_β_5_, or α_5_β_1_ proteins (α_V_β_3_: IT3-H52E3, ACRO Biosystems, Newark, DE, USA; α_V_β_5_: 2528-AV, R&D Systems, Minneapolis, MN, USA; α_5_β_1_: CT014-H2508H, Sino Biological, Beijing, China). For the inhibition group, cyclo(RGDfK) (25 μM, S7834, Selleck, Houston, TX, USA) was preincubated with the integrin protein for 30 min at 37 °C. Next, A 2× binding buffer (100 mM sodium phosphate, 600 mM NaCl, 0.01% Tween 20, pH 8.0) was mixed with the solution at a 1:1 volume ratio and incubated with 5 μL of magnetic beads at 37 °C for 10 min to enable protein immobilization. Following immobilization, the beads were rinsed three times with the binding buffer, and radioactivity was measured using a NaI well-type scintillation counter (2480 Wizard2; PerkinElmer Japan, Yokohama, Japan). Protein binding was expressed as %dose/nmol protein.

### 4.4. Log D and Albumin Binding Measurement

For log D determination, [^125^I]bcRGD, [^125^I]bcRGD_pal_, [^125^I]bcRGD_iba_, or [^125^I]bcRGD_dimer_ (40 kBq) was dissolved in 1.0 mL PBS(–) (pH 7.4) and mixed with 1.0 mL 1-octanol (1:1, *v*/*v*). The mixture was vortexed for 2 min and centrifuged at 5000 rpm for 5 min. Radioactivity in 0.5 mL of each phase was measured using a γ-counter, and log D values were calculated based on the radioactivity distribution (n = 4).

To assess albumin binding, mouse plasma was obtained by centrifugation of whole blood from male ddY mice (9 weeks old). [^125^I]bcRGD, [^125^I]bcRGD_pal_, [^125^I]bcRGD_iba_, or [^125^I]bcRGD_dimer_ (37 kBq) in 50 μL PBS(–) was incubated at 37 °C for 10 min with 200 μL of PBS(–), mouse plasma, or human serum albumin (HSA, 45 mg/mL in PBS(–); Wako Pure Chemical Industries, Osaka, Japan). The mixtures were applied to gel filtration spin columns (Sephadex G-50 Fine; Cytiva, Tokyo, Japan), centrifuged (2000 rpm, 2 min), and radioactivity in the eluted fraction was measured with a γ-counter. The high molecular weight fraction was calculated as the elution ratio = (radioactivity in elution)/(added radioactivity) × 100 (%).

### 4.5. In Vivo Study

#### 4.5.1. Cell Lines

Human glioblastoma U-87 MG cells, generously provided by Prof. Magata (Hamamatsu University School of Medicine), and human lung carcinoma A549 cells, sourced from ATCC (Manassas, VA, USA), were cultured in DMEM supplemented with 10% fetal bovine serum at 37 °C under a humidified 5% CO_2_ atmosphere. To assess α_V_β_3_ and α_V_β_5_ expression, the cells were fixed in 4% paraformaldehyde and incubated at room temperature for 2 h with either an anti-α_V_β_3_ antibody (23C6, 5 μg/mL, Abcam) or an anti-α_V_β_5_ antibody (P1F6, 5 μg/mL, Abcam, Cambridge, UK) as the primary antibody. After washing with PBS(−), the cells were exposed to an Alexa568-labeled secondary antibody (AB4600075, 2 μg/mL, Sigma-Aldrich, St. Louis, MO, USA) for 1 h at room temperature. For nuclear staining, Hoechst 33342 (5 μg/mL, Nacalai Tesque) was applied to the cells for 10 min at room temperature. Fluorescence images were acquired using a BZ-X810 fluorescence microscope (Keyence, Osaka, Japan).

#### 4.5.2. Animal Preparation

Animal experiments were performed in compliance with institutional animal care regulations, with the study protocol approved by the Experimental Animal Committee of Osaka Medical and Pharmaceutical University (Approval Numbers: 21–76, 22–76, and 23–76). Four-week-old male BALB/c nu-nu mice (Japan SLC, Shizuoka, Japan) were housed under a 12 h light/12 h dark cycle with free access to food and water. Tumor-bearing mice were established by suspending U-87 MG cells (2 × 10^6^ cells/mouse) in PBS(–) and subcutaneously injecting 100 μL of the suspension into the right hind leg. For co-implantation models, A549 cells (2 × 10^6^ cells/mouse) were suspended in PBS(–) and inoculated (100 μL) into the left hind leg of the same mice already inoculated with U-87 MG cells. Mice with tumor size approximately 10 mm in diameter, 4–5 weeks after inoculation, were used for biodistribution studies. Animals in which either tumor was not viable were excluded from the experiment.

#### 4.5.3. Biodistribution Study

U-87 MG-bearing mice (n = 42, 8–9 weeks old; 22–27 g) were randomly assigned into groups and administered [^125^I]bcRGD_pal_ or [^125^I]bcRGD_iba_ via intravenous injection (37 kBq in 100 μL of PBS containing 0.1% Tween 80 (PBS-T_80_)). At 5 min, 30 min, 1 h, 2 h, 6 h, 12 h, or 24 h post-injection, the mice were euthanized (n = 3 per time point). The heart, kidneys, liver, brain, pancreas, spleen, lung, small intestine, large intestine, muscle, skin, stomach, bone, and tumors were excised. Each organ’s weight and radioactivity were measured by a NaI well-type scintillation counter, and the % ID/g was calculated. To investigate the correlation between integrin expression and radioactivity accumulation, biodistribution at 2 h after administration of [^125^I]bcRGD_pal_ or [^125^I]bcRGD_iba_ was assessed in A549 and U-87 MG co-implantation mice (n = 5) using the same procedure. Additionally, co-implantation mice were injected with [^125^I]bcRGD_dimer_ (37 kBq/100 μL PBS-T_80_) and euthanized at 30 min, 2 h, and 4 h post-injection (n = 4 per time point), and % ID/g values were calculated as described above. The minimum sample size needed for the biodistribution study in U-87 MG-bearing mice was calculated using G Power software version 3.1.9.7. The calculation was based on a two-tailed test, with an effect size of 3.9, a significance level (α) of 0.05, and a statistical power (1 − β) of 0.8. This analysis indicated that three animals per group were sufficient for the study. The minimum required sample size for the biodistribution study in A549 and U-87 MG co-implantation mice was also calculated to be four animals per group (effect size = 2.5, α = 0.05, 1 − β = 0.8). To guarantee objective results, the experiment was carried out in a blinded manner. Specifically, drug administration and radioactivity measurements were conducted by different investigators to eliminate potential bias.

### 4.6. Statistics

All data are expressed as means ± standard deviation. Statistical analyses were conducted using Tukey’s multiple comparisons test or unpaired *t*-test via GraphPad Prism 8 (GraphPad Software, Boston, MA, USA). Differences were considered statistically significant at the 95% confidence level (*p* < 0.05) unless otherwise indicated.

## Figures and Tables

**Table 1:** Biodistribution of radioactivity following [^125^I]bcRGD_pal_ administration in tumor-bearing mice (% injected dose per gram of tissue).

	Time After Administration						
	5 min	30 min	1 h	2 h	6 h	12 h	24 h
Blood	15.6 ± 1.0	12.2 ± 0.8	9.9 ± 0.5	7.8 ± 0.4	4.5 ± 0.4	2.0 ± 0.3	0.5 ± 0.0
Heart	6.6 ± 0.1	4.7 ± 0.4	4.3 ± 0.3	3.1 ± 0.2	1.7 ± 0.0	0.8 ± 0.1	0.2 ± 0.0
Lung	11.8 ± 1.3	11.2 ± 1.0	9.6 ± 1.3	7.8 ± 1.0	4.4 ± 0.5	2.2 ± 0.3	0.6 ± 0.1
Liver	17.6 ± 0.9	19.0 ± 2.1	15.8 ± 0.9	12.3 ± 0.3	6.0 ± 0.2	1.9 ± 0.2	0.5 ± 0.0
Kidneys	9.0 ± 0.2	8.3 ± 0.9	7.7 ± 0.2	6.7 ± 0.6	4.2 ± 0.6	1.7 ± 0.2	0.4 ± 0.0
Stomach ^¶^	0.5 ± 0.1	0.6 ± 0.1	0.7 ± 0.1	0.8 ± 0.1	0.4 ± 0.0	0.4 ± 0.3	0.1 ± 0.1
Small intestine	2.3 ± 0.2	4.5 ± 0.6	7.0 ± 0.3	9.3 ± 0.9	4.0 ± 0.2	1.9 ± 0.5	0.3 ± 0.0
Large intestine	0.9 ± 0.1	1.4 ± 0.2	1.4 ± 0.1	6.9 ± 1.1	11.8 ± 2.0	7.5 ± 0.8	2.3 ± 1.1
Pancreas	2.8 ± 0.2	2.5 ± 0.3	1.9 ± 0.1	1.6 ± 0.1	1.2 ± 0.3	0.4 ± 0.0	0.1 ± 0.0
Spleen	5.1 ± 0.4	5.9 ± 0.8	4.6 ± 0.6	3.7 ± 0.1	1.9 ± 0.2	0.8 ± 0.1	0.2 ± 0.0
Muscle	1.4 ± 0.3	1.6 ± 0.2	1.4 ± 0.2	1.1 ± 0.1	0.6 ± 0.1	0.3 ± 0.0	0.1 ± 0.0
Bone	1.4 ± 0.1	1.7 ± 0.6	1.1 ± 0.3	1.3 ± 0.4	0.7 ± 0.1	0.4 ± 0.0	0.1 ± 0.1
Brain	0.5 ± 0.1	0.3 ± 0.1	0.3 ± 0.0	0.2 ± 0.0	0.1 ± 0.0	0.1 ± 0.0	0.0 ± 0.0
Thyroid ^¶^	0.1 ± 0.0	0.1 ± 0.1	0.1 ± 0.1	0.1 ± 0.1	0.1 ± 0.0	0.0 ± 0.0	0.0 ± 0.0
U-87 MG	1.4 ± 0.2	3.4 ± 0.5	4.0 ± 0.5	3.9 ± 0.2	2.1 ± 0.4	0.9 ± 0.1	0.3 ± 0.1
U87/Blood	0.1 ± 0.0	0.3 ± 0.0	0.4 ± 0.1	0.5 ± 0.0	0.5 ± 0.1	0.4 ± 0.0	0.5 ± 0.1
U87/Muscle	3.0 ± 0.1	2.1 ± 0.3	2.9 ± 0.2	3.7 ± 0.4	3.3 ± 0.8	3.4 ± 0.5	2.4 ± 0.4

^¶^ Expressed as % injected dose. Data are expressed as mean ± standard deviation (n = 3).

**Table 2:** Biodistribution of radioactivity following [^125^I]bcRGD_iba_ administration in tumor-bearing mice (% injected dose per gram of tissue).

	Time After Administration						
	5 min	30 min	1 h	2 h	6 h	12 h	24 h
Blood	23.3 ± 2.9	18.5 ± 1.0	15.4 ± 1.2	14.0 ± 1.5	8.5 ± 1.0	3.9 ± 0.7	1.4 ± 0.1
Heart	7.4 ± 1.4	5.3 ± 0.5	4.5 ± 0.8	4.2 ± 0.1	2.7 ± 0.2	1.3 ± 0.1	0.5 ± 0.0
Lung	10.7 ± 1.9	9.4 ± 0.3	8.2 ± 0.6	7.5 ± 0.4	5.3 ± 0.5	2.6 ± 0.3	1.2 ± 0.1
Liver	7.6 ± 0.5	6.9 ± 0.2	5.7 ± 0.7	5.1 ± 0.4	3.4 ± 0.4	1.4 ± 0.2	0.6 ± 0.0
Kidneys	7.8 ± 0.5	7.1 ± 1.4	7.3 ± 0.4	5.5 ± 0.3	3.9 ± 0.4	1.8 ± 0.1	0.8 ± 0.1
Stomach ^¶^	0.4 ± 0.1	0.8 ± 0.2	0.7 ± 0.1	0.7 ± 0.3	0.8 ± 0.2	0.2 ± 0.1	0.2 ± 0.0
Small intestine	2.3 ± 0.4	3.0 ± 0.1	4.8 ± 0.6	4.9 ± 0.3	5.6 ± 1.1	2.2 ± 0.5	0.9 ± 0.1
Large intestine	1.5 ± 0.7	1.0 ± 0.1	1.0 ± 0.1	7.1 ± 2.0	11.3 ± 2.0	3.4 ± 1.0	2.2 ± 0.2
Pancreas	2.3 ± 0.4	2.0 ± 0.2	1.5 ± 0.2	1.4 ± 0.2	1.0 ± 0.2	0.5 ± 0.1	0.2 ± 0.0
Spleen	2.9 ± 0.5	3.0 ± 0.2	2.5 ± 0.2	2.3 ± 0.3	1.7 ± 0.5	0.9 ± 0.1	0.4 ± 0.0
Muscle	1.4 ± 0.4	1.5 ± 0.2	1.5 ± 0.1	1.5 ± 0.2	1.2 ± 0.3	0.5 ± 0.1	0.3 ± 0.0
Bone	1.7 ± 0.3	1.9 ± 0.6	1.6 ± 0.4	1.5 ± 0.3	1.3 ± 0.1	0.6 ± 0.2	0.3 ± 0.1
Brain	0.6 ± 0.2	0.4 ± 0.1	0.4 ± 0.0	0.3 ± 0.0	0.2 ± 0.0	0.1 ± 0.0	0.0 ± 0.0
Thyroid ^¶^	0.1 ± 0.0	0.1 ± 0.1	0.1 ± 0.1	0.1 ± 0.1	0.1 ± 0.0	0.0 ± 0.0	0.0 ± 0.0
U-87 MG	2.2 ± 0.5	3.4 ± 0.5	3.6 ± 0.8	3.6 ± 0.7	2.9 ± 0.4	1.4 ± 0.3	0.5 ± 0.1
U87/Blood	0.1 ± 0.0	0.2 ± 0.0	0.2 ± 0.0	0.3 ± 0.0	0.3 ± 0.0	0.4 ± 0.1	0.4 ± 0.1
U87/Muscle	1.6 ± 0.3	2.3 ± 0.6	2.3 ± 0.4	2.4 ± 0.3	2.5 ± 0.3	2.6 ± 0.3	2.1 ± 0.4

^¶^ Expressed as % injected dose. Data are expressed as mean ± standard deviation (n = 3).

**Table 3:** Biodistribution of radioactivity following [^125^I]bcRGD_dimer_ administration in tumor-bearing mice (% injected dose per gram of tissue).

	Time After Administration		
	30 min	2 h	4 h
Blood	1.1 ± 0.1	0.3 ± 0.1	0.2 ± 0.1
Heart	0.6 ± 0.1	0.2 ± 0.0	0.1 ± 0.0
Lung	2.9 ± 0.5	1.2 ± 0.3	0.4 ± 0.1
Liver	0.9 ± 0.1	0.5 ± 0.1	0.3 ± 0.1
Kidneys	10.9 ± 2.6	2.0 ± 0.2	0.9 ± 0.2
Stomach ^¶^	0.6 ± 0.5	0.2 ± 0.0	0.3 ± 0.3
Small intestine	3.6 ± 0.6	2.3 ± 1.0	1.6 ± 0.4
Large intestine	0.4 ± 0.2	5.5 ± 1.0	5.5 ± 1.4
Pancreas	0.5 ± 0.1	0.2 ± 0.1	0.1 ± 0.0
Spleen	0.7 ± 0.2	0.3 ± 0.1	0.2 ± 0.0
Muscle	0.7 ± 0.1	0.2 ± 0.0	0.1 ± 0.0
Bone	1.5 ± 0.3	0.9 ± 0.3	0.4 ± 0.0
Brain	0.1 ± 0.0	0.0 ± 0.0	0.0 ± 0.0
Thyroid ^¶^	0.0 ± 0.0	0.0 ± 0.0	0.0 ± 0.0
U-87 MG	7.5 ± 0.8	4.2 ± 1.1	2.9 ± 0.4
A549	1.7 ± 0.6	0.8 ± 0.2	0.4 ± 0.1
U87/Blood	12.3 ± 4.0	21.8 ± 8.4	18.2 ± 8.0
U87/Muscle	7.1 ± 1.8	13.9 ± 6.8	10.3 ± 2.5

^¶^ Expressed as % injected dose. Data are expressed as mean ± standard deviation (n = 4).

## Data Availability

The data supporting the results and findings of this study are available within the paper and the Supplementary Information Files. Additional raw data are available from the corresponding author upon request.

## Supplementary Materials

The following supporting information can be downloaded at: https://www.mdpi.com/article/10.3390/ph18040549/s1, Figure S1: Chemical structures of peptides; Figure S2: Representative images of fluorescence immunostaining of α_V_β_3_ and α_V_β_5_ in U-87 MG and A549 cells; Figure S3: Radiosynthesis scheme of [^125^I]bcRGD_dimer_; Figure S4: Representative MS spectra for all newly synthesized peptides; Table S1: ESI-MS data of synthetic peptides; supplemental methods.

## Abbreviations

The following abbreviations are used in this manuscript:

PAL	Palmitic acid
IBA	4-(p-iodophenyl)butyric acid
RCY	Radiochemical yield
RCP	Radiochemical purity
HPLC	High-performance liquid chromatography
ESI-MS	Electrospray ionization mass spectrometry
PBS	Phosphate-buffered saline
HSA	Human serum albumin
