# Supplementary material for: Radioiodinated Bicyclic RGD Peptide Derivatives for Enhanced Tumor Accumulation
Source: Pharmaceuticals (Basel). 2025 Apr 8;18(4):549. doi: 10.3390/ph18040549 (PMC12030627; doi:10.3390/ph18040549)
Supplement: Supplementary file 1 [file pharmaceuticals-18-00549-s001.zip › pharmaceuticals-3576655-supplementary.pdf]

Supplementary Information for:

Radioiodinated Bicyclic RGD Peptide Derivatives for Enhanced Tumor Accumulation

N. Kondo *et al.*

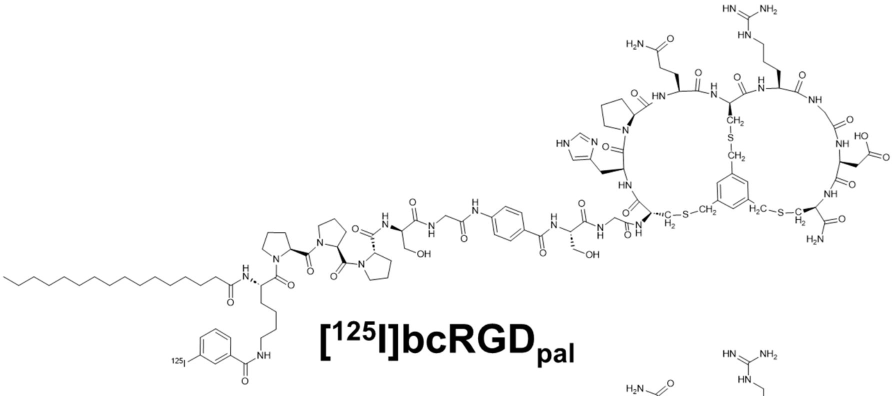

[^{125}I]bcRGD$_{pal}$

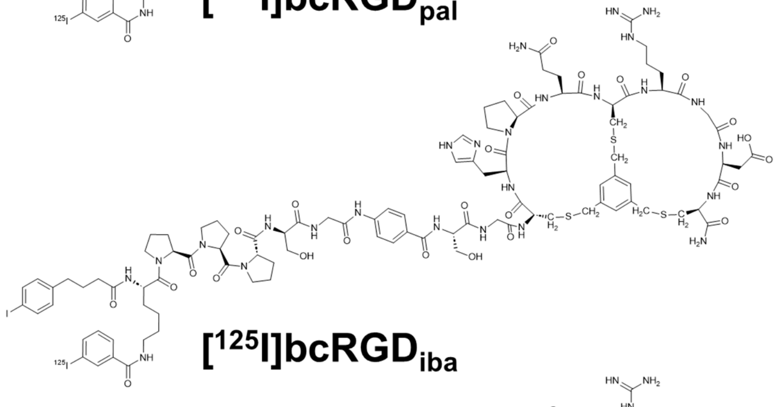

[^{125}I]bcRGD$_{iba}$

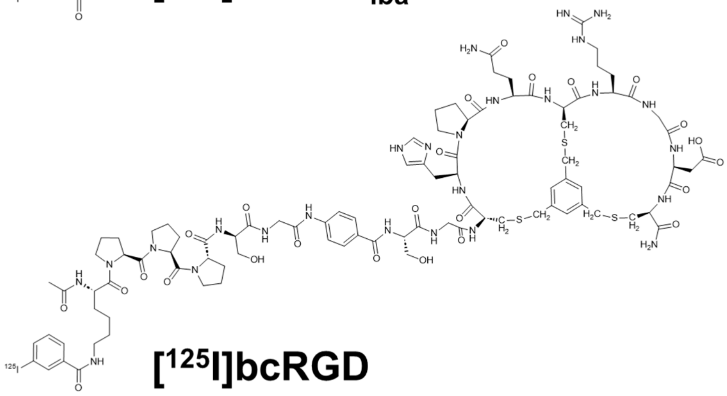

[^{125}I]bcRGD

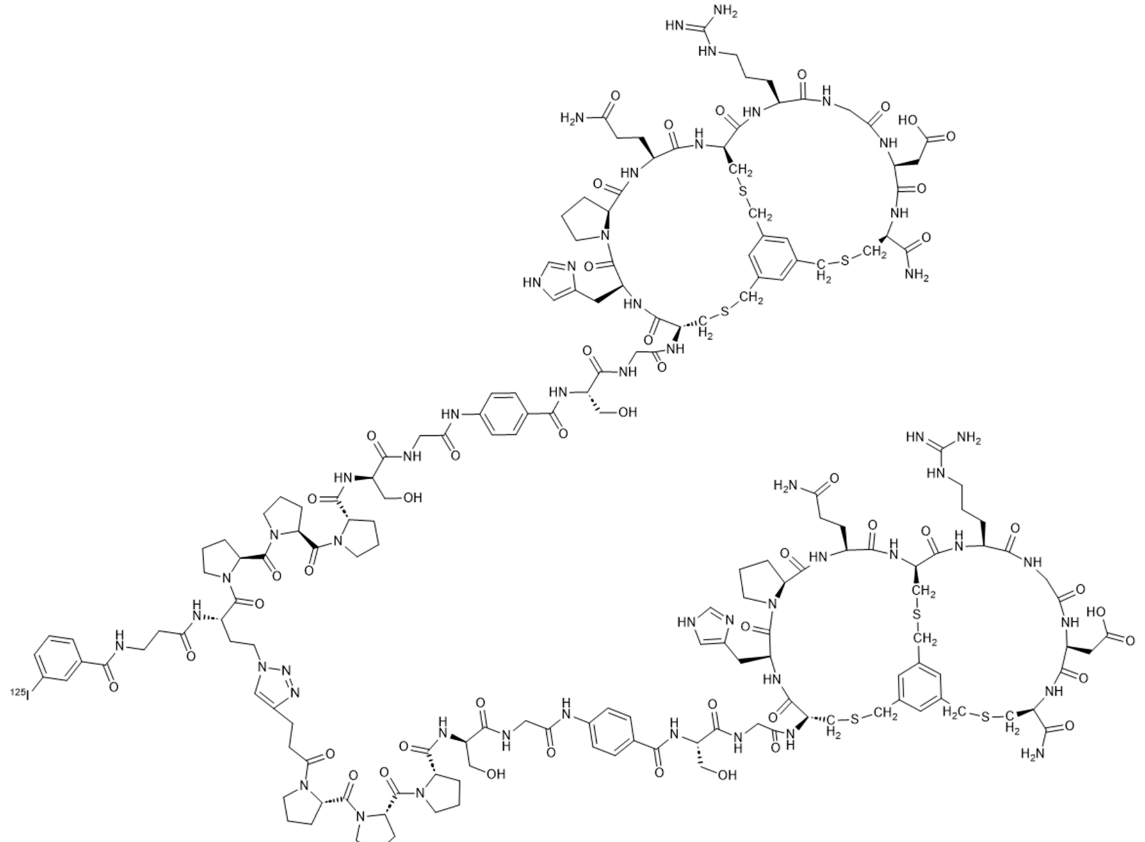

$[^{125}I]bcRGD_{dimer}$

Figure S1

Chemical structures of $[^{125}I]bcRGD_{pal}$, $[^{125}I]bcRGD_{iba}$, $[^{125}I]bcRGD$, and $[^{125}I]bcRGD_{dimer}$

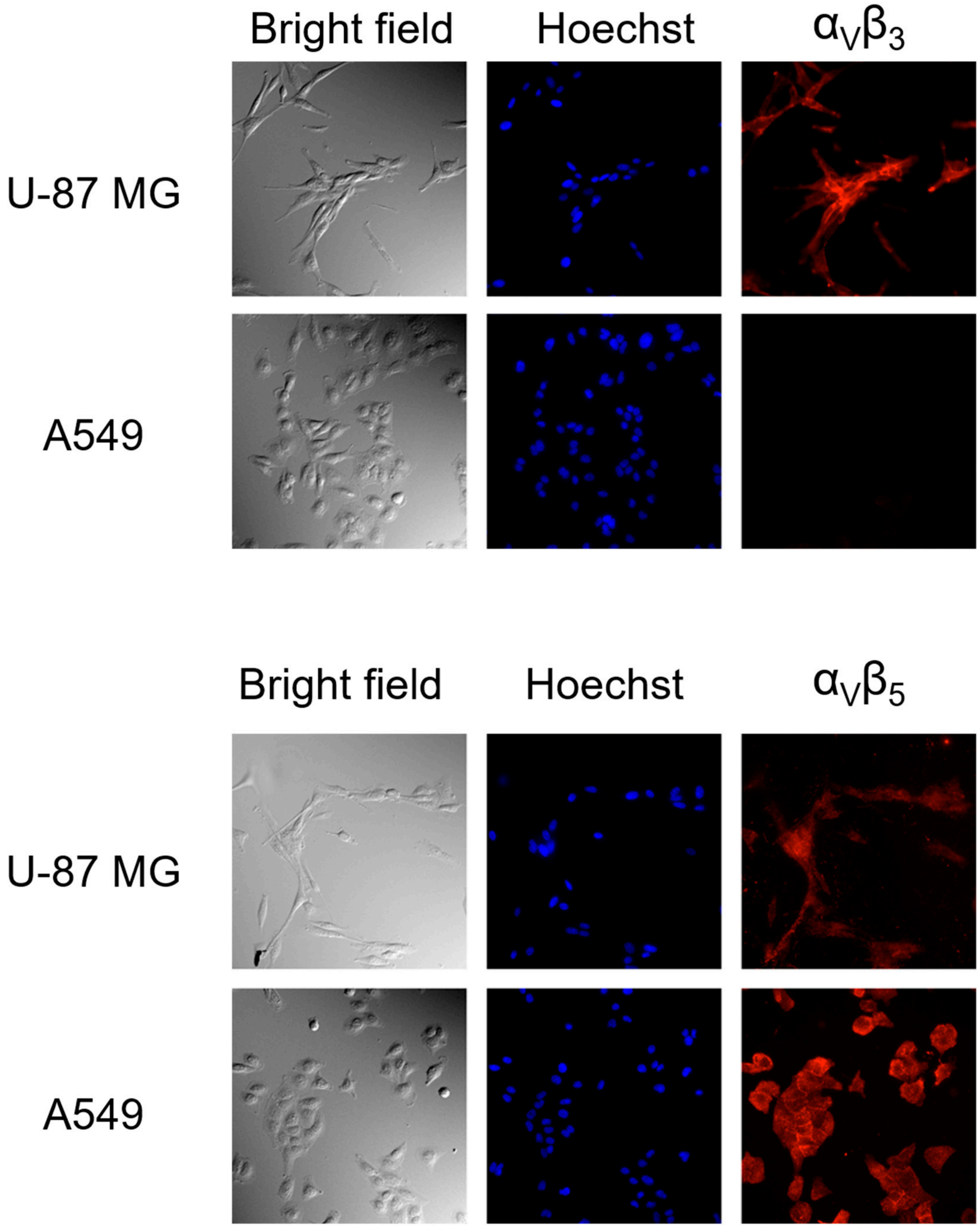

Figure S2

Representative images of fluorescence immunostaining of $\alpha_V\beta_3$ and $\alpha_V\beta_5$ in U-87 MG and A549 cells (Red). Nuclear staining by Hoechst 33342 (Blue).

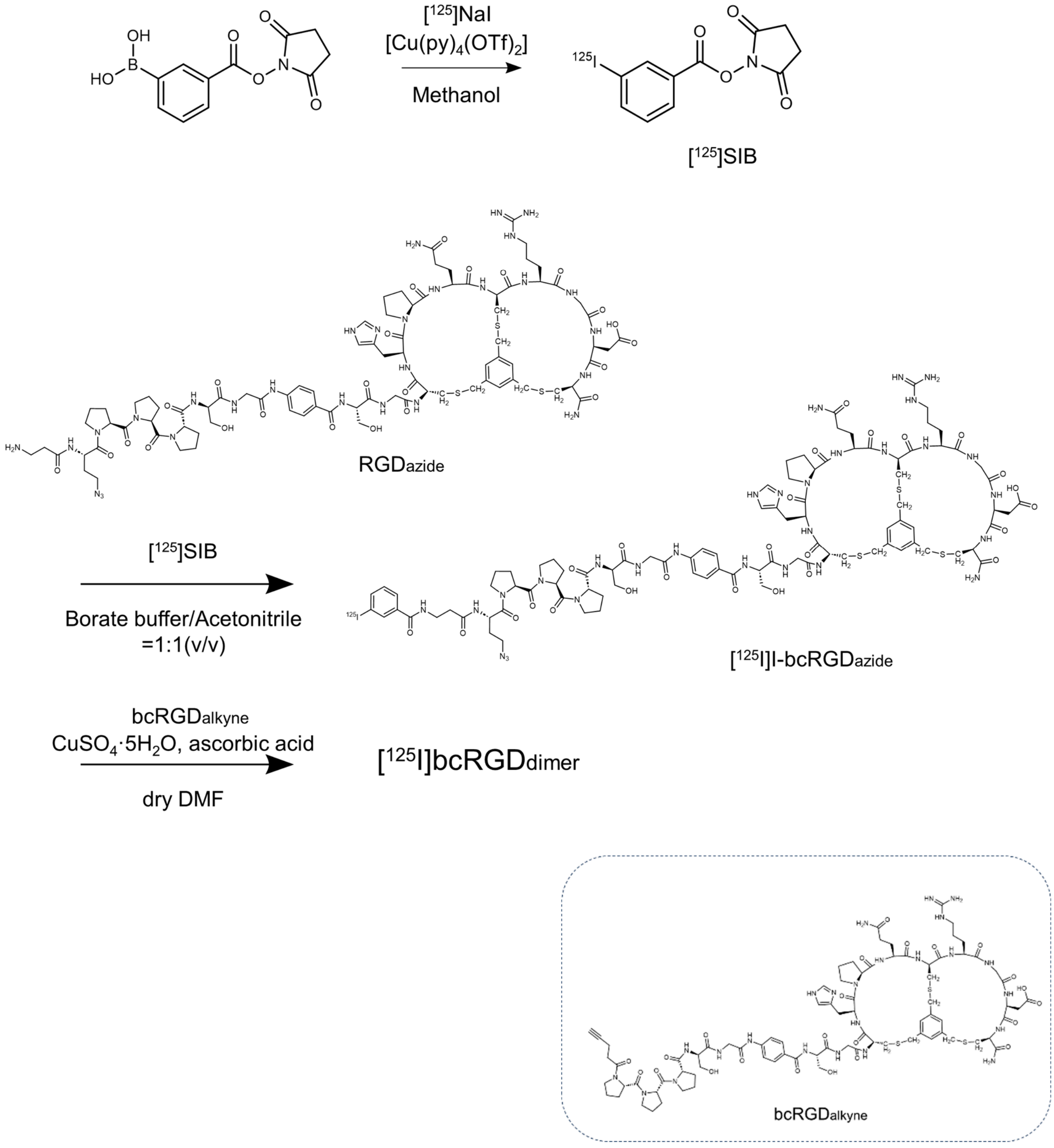

Figure S3

Radiosynthesis scheme of [^{125}I]bcRGD$_{dimer}$

Supplemental Methods

Radiosynthesis of [^{125}I]SIB

m-PB-NHS (200 μg) and Cu(pyridine)$_4$(OTf)$_2$ in Methanol (220 μg/100 μL) were added to a microtube. NaOH aq. solution of [^{125}I]NaI (2 μL, NEZ033L, 25 MBq) was added to the mixture. Then, the tube was vortexed for 15 s. The reaction was left to set at room temperature for 10 min. The product was purified via RP-HPLC, passed through a C18 Sep-Pak cartridge (Waters Corp., MA, USA), washed with water, and eluted with acetonitrile (15–20 MBq/100 μL).

Radiosynthesis of [^{125}I]bcRGD$_{dimer}$

bcRGD$_{azide}$ (200 μg) were dissolved in the mixture of borate buffer (100 μL, 0.1 M, pH 8.5), and the solution was added to [^{125}I]SIB (10-20 MBq) dissolved in acetonitrile (100 μL). After incubation for 1 h at 40 °C, the reaction products were purified by RP-HPLC to yield [^{125}I]bcRGD$_{azide}$. [^{125}I]bcRGD$_{azide}$ was dissolved in dry DMF with $CuSO_4 \cdot 5H_2O$ (2.2 mg), ascorbic acid (3.1 mg), and bcRGD$_{alkyne}$ (200 μg). The mixture was stirred for 1 h at room temperature followed by purification by RP-HPLC.

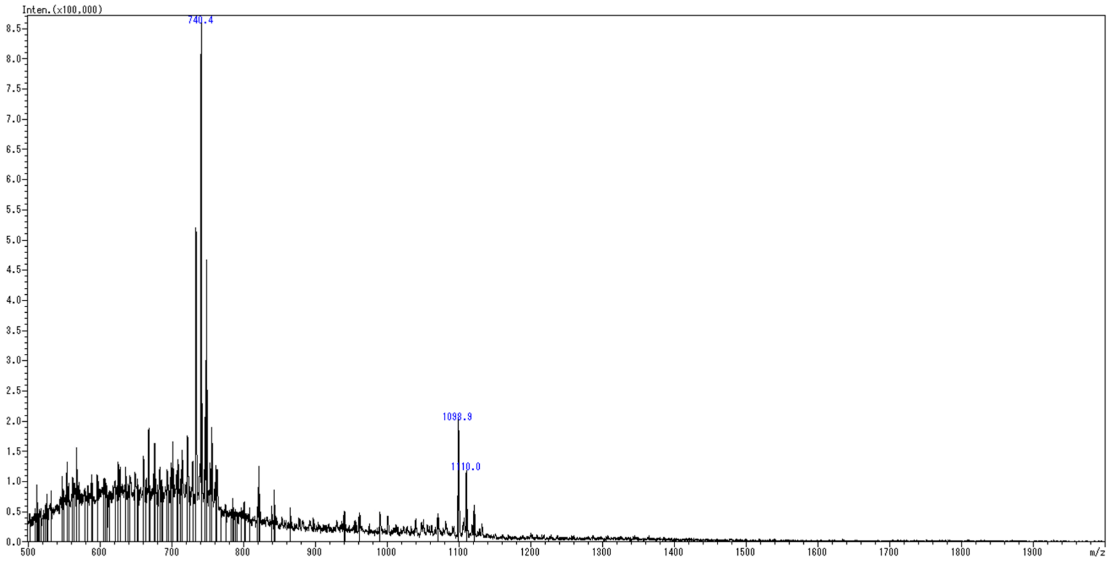

Figure S4a. Representative MS spectrum of $bcRGD_{pal}$

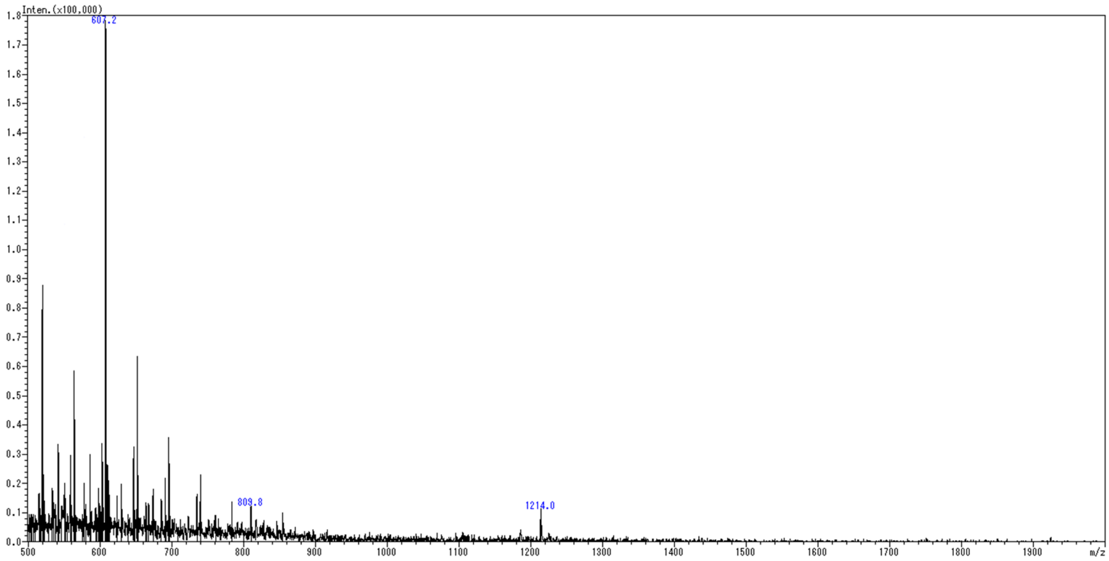

Figure S4b. Representative MS spectrum of I-$bcRGD_{pal}$

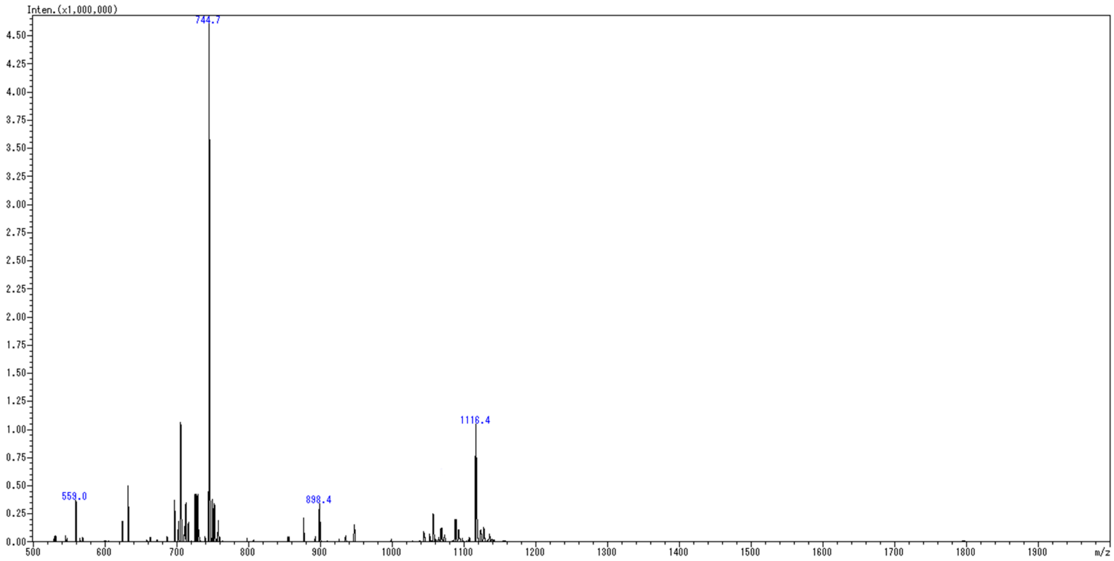

Figure S4c. Representative MS spectrum of bcRGD$_{iba}$

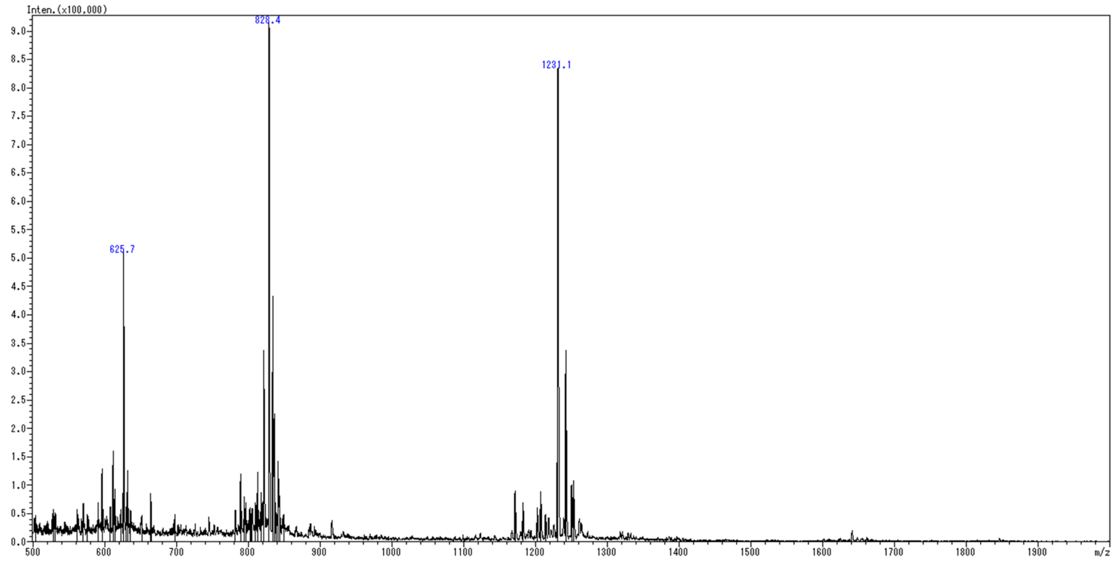

Figure S4d. Representative MS spectrum of I-bcRGD$_{iba}$

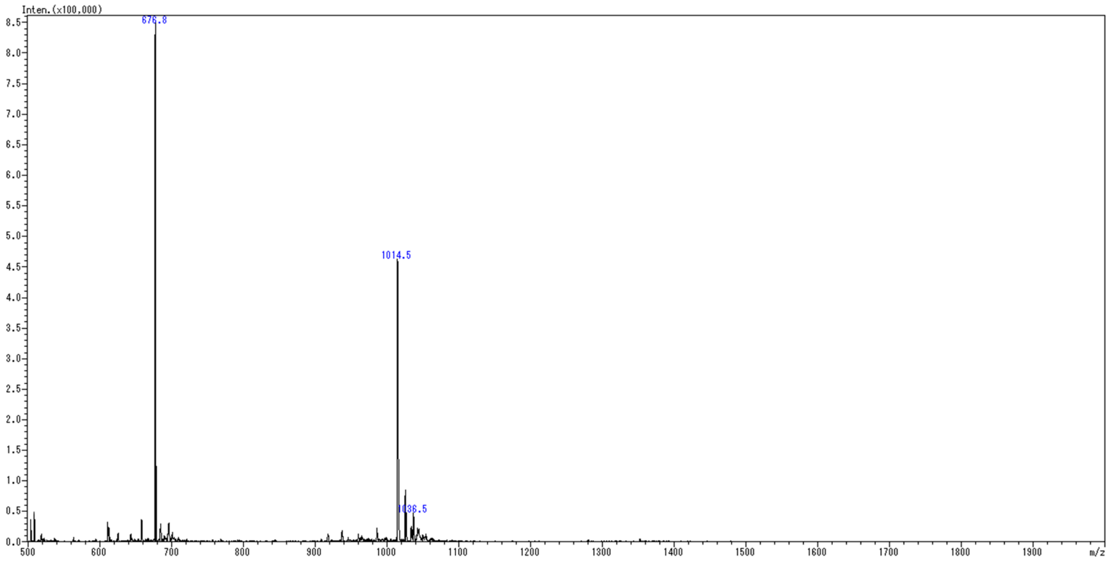

Figure S4e. Representative MS spectrum of $bcRGD_{azide}$

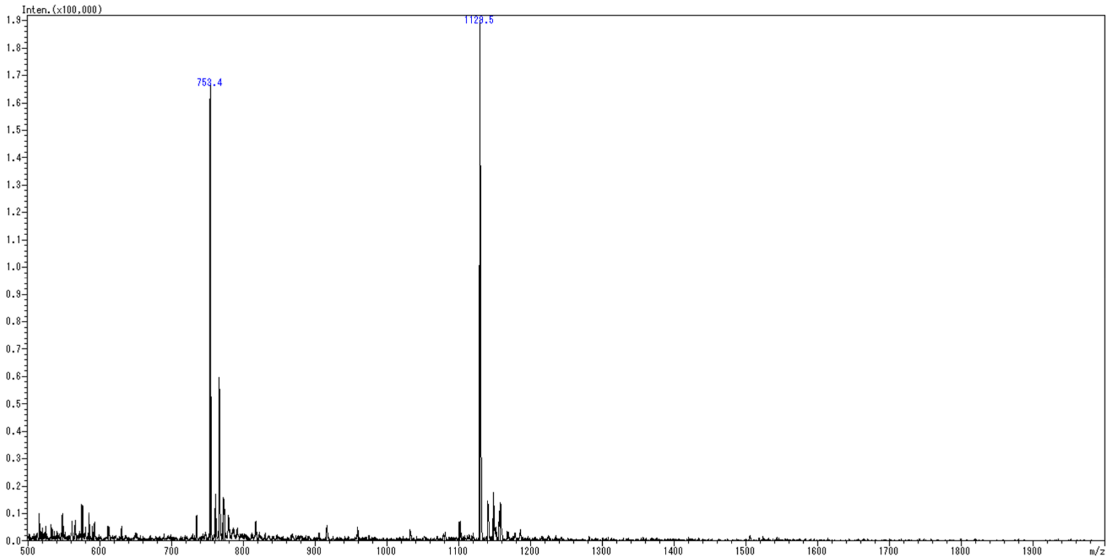

Figure S4f. Representative MS spectrum of I-$bcRGD_{azide}$

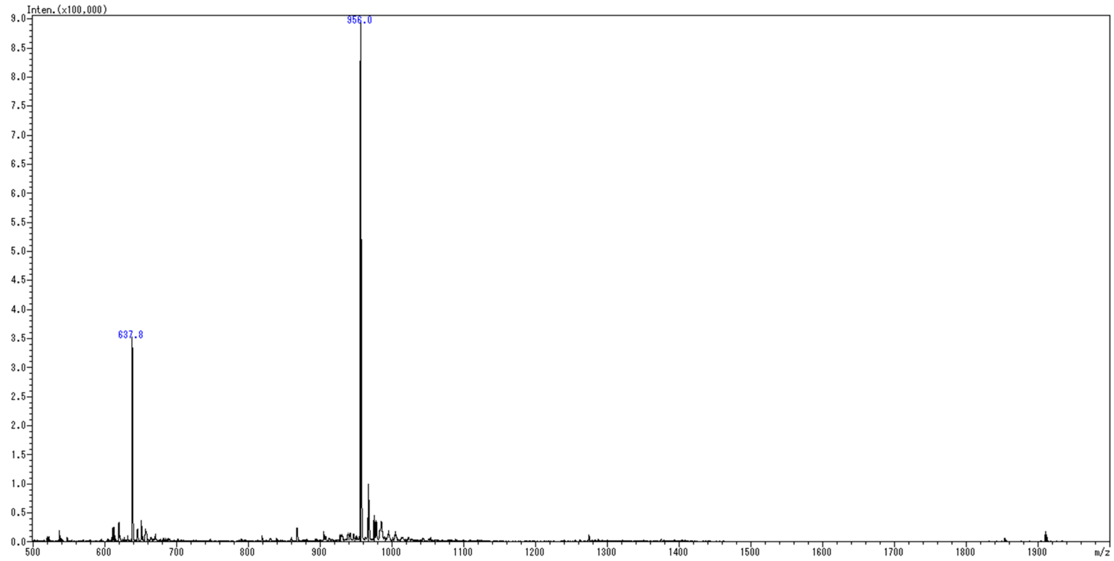

Figure S4g. Representative MS spectrum of $bcRGD_{alkyne}$

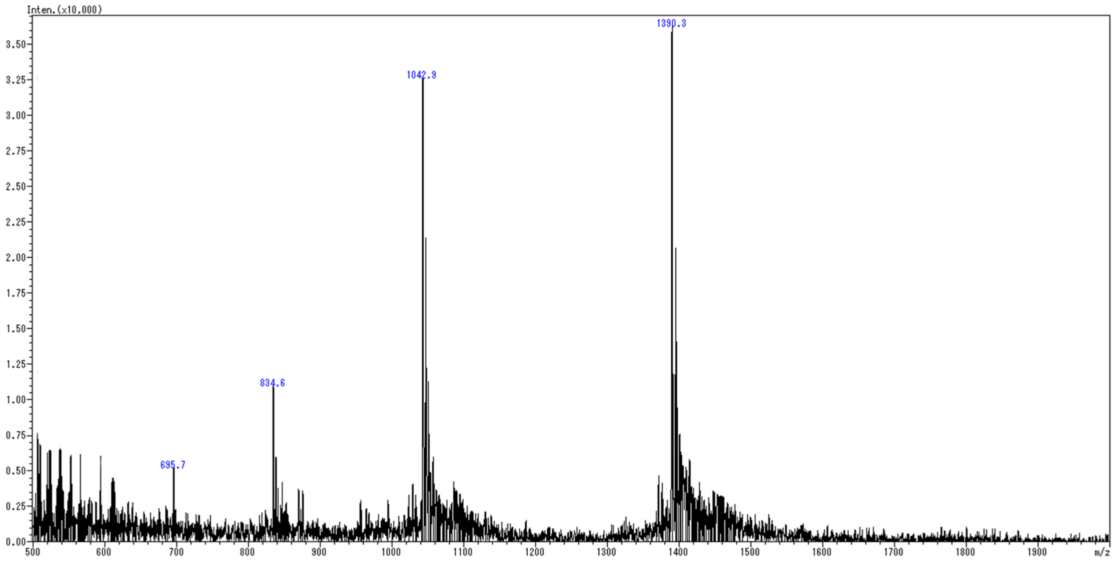

Figure S4h. Representative MS spectrum of $bcRGD_{diner}$

Table S1. ESI-MS data of synthetic peptides.

	Chemical Formula	Calculated *m/z*	Detected *m/z*
bcRGD	$C_{86}H_{122}N_{26}O_{24}S_3$	1999.8	1001.1 $[M+2H^+]^{2+}$, 667.9 $[M+3H^+]^{3+}$
I-bcRGD	$C_{93}H_{125}IN_{26}O_{25}S_3$	2229.8	1115.9 $[M+2H^+]^{2+}$, 744.2 $[M+3H^+]^{3+}$
$bcRGD_{pal}$	$C_{100}H_{150}N_{26}O_{24}S_3$	2196.1	1098.9 $[M+2H^+]^{2+}$, 1110.0 $[M+H^++Na^+]^{2+}$, 740.4 $[M+2H^++Na^+]^{3+}$
$I\text{-}bcRGD_{pal}$	$C_{107}H_{153}IN_{26}O_{25}S_3$	2426.0	1214.0 $[M+2H^+]^{2+}$, 809.8 $[M+3H^+]^{3+}$, 607.2 $[M+4H^+]^{4+}$
$bcRGD_{iba}$	$C_{94}H_{129}IN_{26}O_{24}S_3$	2229.8	1116.4 $[M+2H^+]^{2+}$, 744.7 $[M+3H^+]^{3+}$
$I\text{-}bcRGD_{iba}$	$C_{101}H_{132}I_2N_{26}O_{25}S_3$	2459.7	1231.1 $[M+2H^+]^{2+}$, 828.4 $[M+2H^++Na^+]^{3+}$, 625.7 $[M+2H^++2Na^+]^{4+}$
$bcRGD_{azide}$	$C_{85}H_{119}N_{29}O_{24}S_3$	2026.8	1014.5 $[M+2H^+]^{2+}$, 676.8 $[M+3H^+]^{3+}$
$I\text{-}bcRGD_{azide}$	$C_{92}H_{122}IN_{29}O_{25}S_3$	2256.7	1129.5 $[M+2H^+]^{2+}$, 753.4 $[M+3H^+]^{3+}$
$bcRGD_{alkyne}$	$C_{83}H_{112}N_{24}O_{23}S_3$	1908.7	956.0 $[M+2H^+]^{2+}$, 637.8 $[M+3H^+]^{3+}$
$bcRGD_{dimer}$	$C_{175}H_{234}IN_{53}O_{48}S_6$	4166.5	1390.3 $[M+3H^+]^{3+}$, 1042.9 $[M+4H^+]^{4+}$, 834.6 $[M+5H^+]^{5+}$
